# Chloroplast genome, nuclear ITS regions, mitogenome regions, and Skmer analysis resolved the genetic relationship among *Cinnamomum* species in Sri Lanka

**DOI:** 10.1371/journal.pone.0291763

**Published:** 2023-09-20

**Authors:** Pradeepa C. G. Bandaranayake, Nathasha Naranpanawa, C. H. W. M. R. Bhagya Chandrasekara, Hiruna Samarakoon, S. Lokuge, S. Jayasundara, Asitha U. Bandaranayake, D. K. N. G. Pushpakumara, D. Siril A. Wijesundara

**Affiliations:** 1 Faculty of Agriculture, Agricultural Biotechnology Centre, University of Peradeniya, Peradeniya, Sri Lanka; 2 Postgraduate Institute of Science, University of Peradeniya, Peradeniya, Sri Lanka; 3 Faculty of Engineering, Department of Computer Engineering, University of Peradeniya, Peradeniya, Sri Lanka; 4 Faculty of Agriculture, Department of Crop Science, University of Peradeniya, Peradeniya, Sri Lanka; 5 National Institute of Fundamental Studies, Kandy, Sri Lanka; Georg-August-Universitat Gottingen, GERMANY

## Abstract

*Cinnamomum* species have gained worldwide attention because of their economic benefits. Among them, *C*. *verum* (synonymous with *C*. *zeylanicum* Blume), commonly known as Ceylon Cinnamon or True Cinnamon is mainly produced in Sri Lanka. In addition, Sri Lanka is home to seven endemic wild cinnamon species, *C*. *capparu-coronde*, *C*. *citriodorum*, *C*. *dubium*, *C*. *litseifolium*, *C*. *ovalifolium*, *C*. *rivulorum* and *C*. *sinharajaense*. Proper identification and genetic characterization are fundamental for the conservation and commercialization of these species. While some species can be identified based on distinct morphological or chemical traits, others cannot be identified easily morphologically or chemically. The DNA barcoding using *rbc*L, *mat*K, and *trn*H*-psb*A regions could not also resolve the identification of *Cinnamomum* species in Sri Lanka. Therefore, we generated Illumina Hiseq data of about 20x coverage for each identified species and a *C*. *verum* sample (India) and assembled the chloroplast genome, nuclear ITS regions, and several mitochondrial genes, and conducted Skmer analysis. Chloroplast genomes of all eight species were assembled using a seed-based method.According to the Bayesian phylogenomic tree constructed with the complete chloroplast genomes, the *C*. *verum* (Sri Lanka) is sister to previously sequenced *C*. *verum* (NC_035236.1, KY635878.1), *C*. *dubium* and *C*. *rivulorum*. The *C*. *verum* sample from India is sister to *C*. *litseifolium* and *C*. *ovalifolium*. According to the ITS regions studied, *C*. *verum* (Sri Lanka) is sister to *C*. *verum* (NC_035236.1), *C*. *dubium* and *C*. *rivulorum*. *Cinnamomum verum* (India) shares an identical ITS region with *C*. *ovalifolium*, *C*. *litseifolium*, *C*. *citriodorum*, and *C*. *capparu-coronde*. According to the Skmer analysis *C*. *verum* (Sri Lanka) is sister to *C*. *dubium* and *C*. *rivulorum*, whereas C. *verum* (India) is sister to *C*. *ovalifolium*, and *C*. *litseifolium*. The chloroplast gene *ycf1* was identified as a chloroplast barcode for the identification of *Cinnamomum* species. We identified an 18 bp indel region in the *ycf1* gene, that could differentiate *C*. *verum* (India) and *C*. *verum* (Sri Lanka) samples tested.

## Introduction

The genus *Cinnamomum* Schaeff. of Lauraceae consists of about 247 species (https://powo.science.kew.org/taxon/urn:lsid:ipni.org:names:328262-2#children), found in Asiatic mainland to Formosa, the Malaysian region, northeastern Australia and some Pacific Islands [1,2]. It is a pantropical genus comprised of evergreen trees and shrubs. Biogeographical analysis revealed *Cinnnamomum* originated in the widespread boreotropical paleoflora of Laurasia during the early Eocene (ca. 55 Ma) [3]. *Cinnamomum* species are macromorphologically characterized by buds perulate or not, leaves alternate or opposite, pinnately veined or tripliveined, domatia presence in axil of lateral veins and floral characters such as inflorescences paniculate with cymes bearing strictly opposite lateral flowers. Recent characterization efforts include leaf epidermal micromorphology, having a reticulate periclinal wall or non-reticulate periclinal wall and pollen morphology [4,5].

*Cinnamomum verum* J. Presl (Synonymous with *C*. *zeylanicum* Blume), *C*. *aromaticum* Nees and *C*. *camphora* (L.) J. Presl (= *Camphora officinarum* Nees) are commercially traded worldwide, while several other species such as *Cinnamomum burmannii* (Nees & T. Nees) Blume (Indonesian cinnamon) and *Cinnamomum loureiroi* Nees (Vietnamese cinnamon) [6], and *C*. *tamala* T. Nees and Eberm (India and Nepal) [7] also have some economic benefits. *Cinnamomum verum*, recognized as Ceylon cinnamon or true cinnamon in the world market, has recently gained special attention due to scientific evidence of its medicinal benefits [8,9]. Historical evidence suggests that *C*. *verum* has first been identified in the natural rainforests in the upcountry region of Sri Lanka and brought to cultivation after 1500 AC [10]. Apart from that, Sri Lankan rainforests are home to seven endemic wild species of cinnamon. They are *C*. *capparu-coronde* Blume, *C*. *citriodorum* Thwaites, *C*. *dubium* Nees, *C*. *litseifolium* Thwaites, *C*. *ovalifolium* Wight, *C*. *rivulorum* Kosterm, and *C*. *sinharajaense* Kosterm [11,12]. Among them, species such as *C*. *sinharajaense*, *C*. *rivulorum* and *C*. *dubium* are restricted to specific environments, while others such as *C*. *litseifolium* and *C*. *ovalifolium* are naturally grown in several agro-ecological zones. The morphology of some species drastically changes when they are grown or cultivated under other agroecological conditions [13,14]. In addition, the natural cross-pollination behavior [5,15] of *Cinnamomum* has created considerable intraspecies diversity [16], reflected by both morphological and biochemical traits [17]. Therefore, morphology based identification of *Cinnamomum* species is challenging [3,18–20].

Molecular biological tools have been used for studying the inter-species diversity of several *Cinnamomum* species, including a few in Sri Lanka [21–33]. Among them, few early studies depended on PCR amplification and sequence analysis, Randomly Amplified Polymorphic DNA (RAPD), and sequence-related amplified polymorphism (SRAP) [23,33]. While some authors could fully resolve the phylogeny of *Cinnamomum* species [23], others could not reach the expected results [33]. DNA barcoding has also been successfully used for molecular identification of some *Cinnamomum* species [34–37]. However, recent work showed that the universal barcoding regions *rbc*L, *mat*K, and *trn*H*-psb*A do not have sufficient polymorphism for the clear identification of *Cinnamomum* species found in Sri Lanka [25]. Similarly, several other authors have also showed that chloroplast genes, *trn*L-*trn*F, *trn*T-*trn*L, *psb*A-*trn*H, *rp*l16, *mat*K, and nuclear DNA were not powerful to resolve the phylogenomic relationships of Lauraceae family members [38–41]. Nevertheless, correct species identification is critical for conservation and sustainable utilization and industrial applications.

Chloroplast genome is used as an ultra-barcode in recent phylogenomic studies because of the advancement of next-generation sequencing technology, the improvement of sequence assembly software, and the reduction of sequencing costs [34–36]. Scientists have assessed both coding and non-coding regions of the plastome [42]. In addition, the nuclear ribosomal Internal Transcribed Spacer regions (ITS) are also utilized widely [43]. Skmer is a sample separation tool that uses genome skimming data without assembling or aligning sequences [44]. Illumina data are utilized in recent studies for assembling both chloroplast and mitochondrial regions [45]. Therefore, we utilized 20x coverage Illumina Hiseq data to assemble chloroplast genomes, a few mitochondrial regions, nuclear ITS regions, and Skmer analysis to resolve the genetic relationship among endemic *Cinnamomum* species in Sri Lanka. We also included several *C*. *verum* samples from India and publicly available sequencing data in the analysis. We carefully assessed the data to identify a suitable region for PCR-based identification of closely related *Cinnamomum* species.

## Materials and methods

Our analysis includes the chloroplast genome, ITS regions, several mitogenome regions, and the Skmer procedure including randomly selected 500,000 reads of all *Cinnamomum* species present in Sri Lanka. The wet lab data were generated to confirm some results of bioinformatics analysis. We present the method under subtopics including the relationship among considered species based on our data and the publicly available sequencing data.

### Sample collection, DNA extraction, sequencing

#### Sample collection

The Research Committee of the Department of Wildlife Conservation, Sri Lanka, and the Department of Forestry, Sri Lanka, granted permission to collect wild *Cinnamomum* species from the rainforests in Sri Lanka and the permission was granted from the Department of Export Agriculture to collect cultivated *C*. *verum* (Sri Lanka) samples from the respective research stations. Furthermore, all the samples were collected according to relevant institutional, national, and international guidelines and legislation.

Nine cultivated *C*. *verum* (Sri Lanka) accessions previously identified as different from each other based on morphological and biochemical traits [17] were collected from a vegetatively propagated plantation at Nillambe, Sub Research Station, and germplasm collection at the National Cinnamon Research and Training Center, Thihagoda, Palolpitiya Department of Export Agriculture (DEA).

Seven endemics wild *Cinnamomum* species were collected from the rainforests, and the germplasm collections at the National Cinnamon Research and Training Center, Thihagoda, Palolpitiya, and Mid Country Research Station, Dalpitiya, DEA. The identity of the collected wild samples was verified using typical morphological characters by Siril A. Wijesundara. Sample collection details are given (S1 Table). The collected specimens were further verified with the voucher specimens at the National Herbarium, (PDA), the Royal Botanic Gardens, Peradeniya. The standard herbarium specimens were prepared by mounting on herbarium sheets and deposited at the National Herbarium (PDA) the Royal Botanic Gardens, Peradeniya as previously described [25]. Some morphological, biochemical, and molecular traits of considered samples are included in recent publications [16,19,20,25].

In addition, we included two authentic *C*. *verum* (India) bark samples and a market sample from India in the analysis. Altogether, a total of twenty *Cinnamomum* samples, ten *C*. *verum* (Sri Lanka), seven wild species and three *C*. *verum* (India) samples were included in the analysis.

#### DNA extraction

Total genomic DNA from all *C*. *verum* (Sri Lanka) samples and *C*. *verum* (India) was extracted using Promega Wizard® Genomic DNA Purification kit (Cat. No: A1120) following the manufacturers’ guidelines. Total genomic DNA from all the wild samples was extracted using the Cetyltrimethylammonium bromide (CTAB) method [46] with modifications as previously described [25,47]. The extracted DNA samples were re-suspended in 50 μL nuclease-free water, quantity and quality assessed with a NanoDrop spectrophotometer (NanoDrop 2000, Thermo Scientific) and running on 0.8% agarose gels before storing at 4°C.

### Chloroplast DNA sequencing

A total of 1 μg DNA from each sample was sent to Admera Health, USA, for chloroplast genome sequencing using Illumina Hiseq with 20-30x coverage per sample. The DNA quality and quantity were tested with a Qubit fluorometer (ThermoFisher) and TapeStation system (Agilent). The DNA Libraries were prepared using KAPA Hyper Prep kit (Roche, Switzerland) and sequenced on an Illumina Hiseq platform with a read length of 2 x 150 bp paired-end while giving 80–90 M paired-end reads per sample.

### Chloroplast genome assembly and analysis

#### Chloroplast genome assembly

Using NOVOPlasty assembler (version 2.7.2) [40] we first assembled each dataset into contigs. The assembler used *Zea mays* chloroplast gene of the large subunit of RUBP (V00171.1) as a seed to jump-start the assembly. This method extends the seed iteratively in both directions by adding overlapping reads from the dataset.

We configured assembly parameters such as the insert size and read length of the dataset according to sequencing specifications, and set the kmer size to 25. As per the assembler developer’s instructions, we only trimmed the adapters from the raw data and did not filter any low-quality reads before assembly. For each dataset, NOVOPlasty filtered the chloroplast reads from the total DNA and generated a set of contigs. We imported each set of contigs into Geneious (version 11.0.5) [48] and assembled them into a consensus sequence using the ‘*De novo* assembly’ option and built-in Geneious assembler.

#### Annotation

We used the GeSeq web [49] annotation tool to annotate the chloroplast genomes. Selected annotation options for a given fasta file included ‘Annotate plastid IRs’ and ARAGORN software [50] for *de novo* tRNA annotation. As references for BLAST search, we selected the three taxa from the genus *Cinnamomum* from the NCBI nucleotide database: *C*. *camphora* (NC_035882.1), *C*. *micranthum* (NC_035802.1), and *C*. *verum* (NC_035236). The web tool generated several output files including the GenBank format of the annotation and the GFF3 file. When provided with the corrected GenBank file as input, the OGDRAW web tool (https://bio.tools/ogdraw) produced a graphical annotation map of each species.

Considering that the *C*. *verum* (India) sample was a bark while all other samples were leaves, it was necessary to determine whether there was sufficient chloroplast DNA within the total DNA of all samples. For that, we mapped the total genomic raw reads of each sample to its seed-based chloroplast assembly with the Bowtie2 [51] program embedded in Geneious. The number of mapped reads out of total reads, maximum coverage, and mean coverage of reads were the statistics recorded.

#### Chloroplast sequence alignments and phylogenomic analysis

A total of 82 complete chloroplast genomes, which included nine *Cinnamomum* samples sequenced and assembled in this study and 73 complete chloroplast data deposited in NCBI, representing 26 species of *Cinnamomum*, were aligned using MAFFT v7.450 (algorithm FFT-NS-2) built-in Geneious (version 11.0.6) [52].

The Geneious plugin program MrBayes v3.2.6 [53] was used to perform a phylogenomic analysis. MrBayes is a program for Bayesian inference that uses Markov chain Monte Carlo (MCMC) methods to estimate the posterior distribution of model parameters. Under the default settings, the MCMC process was run for 1,100,000 and the first 100,000 generations were discarded as burn-in. The remaining raw trees were sub-sampled at a frequency of 200 to build a consensus tree to represent the summary of the samples. Then the statistical support for each branch of the consensus tree was calculated using the frequencies of the sampled trees in which a particular branch appeared. Bayesian Posterior Probability (BPP) was calculated as the proportion of sampled trees that contain a specific branch, out of the total number of sampled trees. Finally, the majority-rule consensus tree was generated by grouping the strongly supported branches together. *Ocotea porosa* was selected as the out group because *Ocotea* is one of the largest genera in the Lauraceae (400 spp.) and it has been known to be paraphyletic with respect to most other genera of the New Word Lauraceae for almost 20 years [54,55]. Finally, the majority-rule consensus tree was generated using the raw trees, sub-sampled at a sample frequency of 200^th^ iterations.

#### Nucleotide diversity analysis

The alignment was examined for nucleotide diversity among the *Cinnamomum* species using the DnaSP v6.12.03 [56] DNA polymorphism analyzer. Nucleotide variability (Pi) was calculated using a sliding window method (window length 600 bp and step size 200 bp).

#### Simple Sequence Repeat (SSR) analysis

To find perfect Simple Sequence Repeats (SSR), Krait v1.3.3 [57] was used with the minimum number of repeats set to 8, 4, 3, 3 and 3 for mono-, di-, tri-, tetra-, and pentanucleotide SSRs, respectively.

### ITS regions assembly

#### ITS regions extraction

First, we extracted nuclear reads by mapping the total DNA sequence reads of each species to the assembled chloroplast genome using Bowtie2 [51]. The non-mapped reads were considered the nuclear reads assuming the number of reads from the mitochondrial genome was negligible.

The time complexity of SPAdes is proportional to the size of the input data and can be estimated as O (N log N), where N is the total size of the input reads [58]. However, the actual time complexity of SPAdes may be much higher in practice, especially for large and complex genomes or datasets with high levels of sequencing coverage. Therefore, considering the computational time and the RAM capacity required to assemble the reads using SPAdes [58], we randomly selected 5GB reads for the contig assembly. Then, the ITSxpress [59] was executed, taking the assembled contigs as the input. The output sequences were taken as the candidate ITS regions for each *Cinnamomum* species. Each assembled ITS sequence contains an 18S ribosomal RNA gene (rRNA) (Partial sequence), ITS 1, 5.8S rRNA, ITS 2, and 26S rRNA (partial sequence). ITS region for a *C*. *verum* sample was extracted from the raw data deposited in the NCBI (SRX2990994) [60].

#### ITS regions validation

We validated the obtained ITS regions using NCBI Blastn with default parameters. The BLAST results for each species ITS regions indicated 100% query coverage and more than 99% per identity for *Cinnamomum verum*. Further, we computationally validated the obtained ITS regions using the ITS2 annotator [61]. This tool uses HMMer to annotate ITS2 regions of eukaryotes with Hidden Markov Models (HMMs). It returns the sequence between the conserved 5.8S and 28S (or 26S) rRNA according to the ITS2 definition. We annotated ITS2 sequences of each ITS region, and the results confirmed that the assembled ITS regions are adequate for further analyses.

#### ITS Sequence alignment and phylogenomic analysis

We carried out a multiple sequence alignment for extracted ITS regions (18S ribosomal RNA gene (rRNA) (Partial sequence), ITS 1, 5.8S rRNA, ITS 2, and 28S rRNA (partial sequence) for 10 *Cinnamomum* samples, including nine *Cinnamomum* samples from this study and one from NCBI. Furthermore, we compared the ITS regions of nine *Cinnamomum* samples with additional sequences retrieved from NCBI. Considering the sequence data availability and query coverage, we downloaded sequence data of ITS 1, 5.8S rRNA, and partial sequence of ITS 2 region for additional 38 samples representing 17 *Cinnamomum* species. A total of 50 samples were subjected to multiple sequence alignment, and Bayesian phylogenomic analysis was carried out as mentioned under the above chloroplast sequence alignments and phylogenomic analysis using Geneious (version 11.0.6). *Ocotea porosa* (MF110078, MK507282) was selected as the out group.

#### Mitochondrial genes–assembly and analysis

The mitochondrial genes *atp1*, *atp6*, and *cox1* are highly polymorphic in *Silene vulgaris*, [62]. Additionally, *matR* and *atpA* genes are also used in plant phylogeny work [63]. Therefore, we included these mitochondrial genes in our analysis. Initially, we downloaded the *C*. *camphora atp1* gene (AF197681), *Laurus nobilis atp6* gene (AY831985), and *C*. *verum cox1* gene (AY009440), *C*. *camphora matR* gene (AF197797) and *C*. *verum atpA* gene (AY009415) from NCBI. We mapped the raw reads of nine *Cinnamomum* samples to the reference gene sequences using a custom script (https://github.com/AgBC-UoP/mapNclean-nf). This script uses Bowtie2 v2.3.3.1 to align reads and samclip v0.4.0 (https://github.com/tseemann/samclip) to remove clipped reads. Consensus sequences for the read alignments were generated using the Geneious prime software followed by manual adjustment to remove ambiguity codes. During the alignment, any ambiguous bases in our consensus sequences were identified and replaced with the respective base observed in the majority of the samples. Specifically, if a particular nucleotide base was present in at least 80% of the other samples or if every other sample had the same nucleotide base, we replaced the ambiguous base in our consensus sequence with that nucleotide base. The same regions for a *C*. *verum* sample were extracted from the raw data deposited in the NCBI (SRX2990994) (60). Then we generated a Neighbor-Joining Consensus Tree for a combined data set of *atp6* (631 bp) and cox*1* (1415 bp) for 10 *Cinnamomum* species with the Tamura–Nei genetic distance model and 100 bootstrap replicates for node supports. The *atp6* (631 bp) and cox*1* (1415 bp) genes were selected for the Neighbor-Joining Consensus tree construction as they have variable sites among the studied mitochondrial genes. Since there was no publicly available convincing data for selected genes for other *Cinnamomum* species except for *C*. *verum* (SRX2990994), only the previously generated *C*. *verum* data (SRX29990994) was included in this analysis.

#### Skmer analyses

Skmer analysis is a computational technique used for analyzing genomic or metagenomic sequence data [44]. It involves breaking up the sequence data into overlapping k-mers, and then counting the frequency of occurrence of each k-mer in the dataset. The resulting k-mer frequencies can be used to generate an unrooted tree. Here we used the entire skimming dataset of a species without separating reads into nuclear, chloroplast or mitochondrial DNA.

Only 500,000 reads were randomly extracted from each forward and reverse fastq file for each*Cinnamomum* skimming dataset. Then the forward and reverse reads were concatenated to form subsamples of 1,000,000 reads. Then, Skmer v3.0.2 [44] was used to perform an assembly-free and alignment-free c analysis of *Cinnamomum* samples. Only the datasets generated in the current study were included in the analysis since similar *Cinnamomum* data sets were not available on the public domain.

### Primer designing, PCR and Sanger sequencing to amplify highly variable regions in chloroplast

#### PCR amplification of chloroplast *ycf1* gene regions

Two primer pairs (Table 1) were designed to amplify two regions of the *ycf1* gene identified as highly variable regions in the chloroplast genome based on the nucleotide diversity analysis described above. The PCR amplification was carried out for 10 *C*. *verum* samples, eight from Sri Lanka and two from India. A total of 30 μL contained lx PCR buffer, 1.5 mM MgCl_2_, 200 mM dNTP (Promega, USA), 0.2 μM of each primer (Integrated DNA Technologies, Singapore), 100 ng of DNA, 0.8 μM spermidine, and 1 Unit Go Taq Flexi DNA polymerase (Promega, USA). The PCR cycle consisted of initial denaturation at 94°C for 2 minutes, followed by 35 cycles of 94°C for 1 minute, annealing at 48°C for 30 seconds, elongation at 72°C for 30 seconds, and a final extension at 72°C for 3 minutes.

**Table 1:** Primers used to amplify highly varying regions of the chloroplast genome.

Primer	Sequence 5’ to 3’	Product size bp
Primer set 1	ATGTCCGATTCCGTGTAATC TTATCTCAGGCTAGGTGGTC	952
803 *ycf1* f2 1755 *ycf1* r2		
Primer set 2	TCTGCTATTGCTATCCGTTC ATGCTTGCCTAGAGTGTATG	992
3203 *ycf1* f5 4195 *ycf1* r5		

Products were separated by electrophoresis (5 Vcm^-1^) on 1.5% agarose gels and stained with safe green (Applied Biological Materials Inc. Canada). The PCR products were shipped to Macrogen Inc (Seoul, South Korea– http://dna.macrogen.com) for Sanger sequencing using the same primers as used for PCR.

### *ycf1* gene regions sequence alignments and analysis

Chromatograms of the PCR amplified products of highly variable regions were visually inspected using Geneious for sequencing errors, and the 5’ and 3’ noisy sequences of about 30 bp were removed. The same regions of the seven wild *Cinnamomum* species, *C*. *verum* (India) and *C*. *verum* (Sri Lanka) were extracted from the assembled chloroplast genomes.

Sequences of the PCR amplified two *ycf1* gene regions were extracted for *C*. *aromaticum* (NC_046019) and *C*. *verum* (NC_035236) samples using the chloroplast sequences deposited in the GenBank. Each region was aligned using Geneious alignment of Geneious Prime Software. The ends were trimmed and joined 846 bp and 794 bp of each region. Sequence divergence of *C*. *verum* samples collected from Sri Lanka and India were calculated using the Tamura-Nei model of Molecular Evolutionary Genetics Analysis (MEGA-X) software [64,65].

## Results

We present the results under subtopics parallel to the methodology including the relationship among considered species based on our data and the publicly available sequencing data.

### Chloroplast genome assembly and analysis

#### Chloroplast genome assembly

For the chloroplast seed-based assembly using NOVOPlasty software, data of all species were assembled with consistency (Table 2). Assembly length for all nine assemblies ranged between 152695 bp and 152797 bp without drastic deviations. *Cinnamomum capparu-coronde* was the smallest chloroplast genome (152695 bp), while *C*. *sinharajaense* was the largest one (152797 bp). The size of the assembled *C*. *verum* chloroplast genome of the samples from Sri Lanka (152765 bp) is very close to the chloroplast genome of *C*. *verum* published in NCBI (NC_035236), which is 152766 bp long.

**Table 2:** Seed-based assembly statistics for nine *Cinnamomum* samples.

Species (GenBank acc. no.)	Total reads	Assembled reads	% of assembled cp reads	Number of contigs	N50	Assembly length (bp)	GC%
*C*. *capparu-coronde* (ON685904)	112428684	737058	0.66	3	133833	152695	39.1
*C*. *citriodorum* (ON685905)	93305002	811122	0.87	3	133892	152714	39.1
*C*. *dubium* (ON685906)	167974904	1895208	1.13	3	133881	152785	39.1
*C*. *litseifolium* (ON685907)	108723790	685568	0.63	3	133869	152745	39.1
*C*. *ovalifolium* (ON685908)	119032250	495094	0.41	3	133868	152708	39.1
*C*. *rivulorum* (ON685909)	95762972	781514	0.82	3	133873	152787	39.1
*C*. *sinharajaense* (ON685910)	102394356	2540352	2.48	3	133920	152797	39.1
*C*. *verum* (India) (ON685911)	107469622	581954	0.54	6	68777	152727	39.1
*C*. *verum* (Sri Lanka) (ON685912)	129226378	1649924	1.28	3	133852	152765	39.1

Further, N50 values for all assemblies were comparable with previous data [66–70], while *C*. *verum* (India) has a smaller N50 value among the nine (9) chloroplast assemblies than the rest. Out of total DNA, more than 0.4% of chloroplast reads were assembled for all datasets of all nine (9) species.

#### Genome annotation

**Fig 1:**
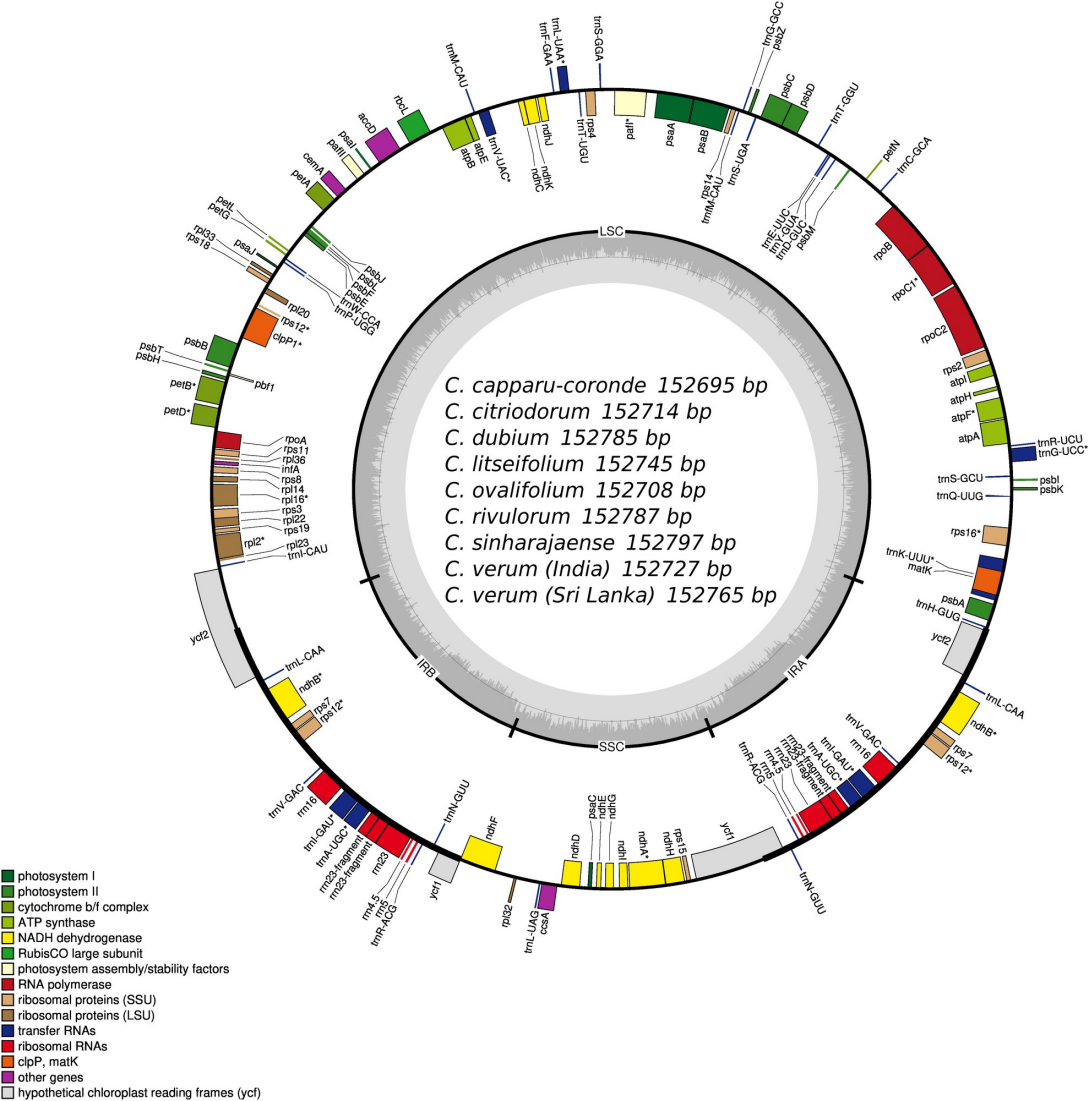
Annotation of draft chloroplast genomes of nine *Cinnamomum* species.

With GeSEQ and OGDRAW web tools, we were able to annotate all nine (9) chloroplast genomes and visualize the chloroplast genome annotations (Fig 1).

The typical angiosperm chloroplast genome consists of 4 rRNAs, approximately 30 tRNAs, and 80 protein coding genes in its gene content [71]. All *Cinnamomum* assemblies included 36 tRNA genes in each assembly (S2 Table).

#### Chloroplast sequence alignments and phylogenomic analysis

The distance matrix obtained for the multiple sequence alignment of all 82 *Cinnamomum* samples and *O*. *porosa* is given in S3 Table. There are only five (5) variable sites between *C*. *v*erum (Sri Lanka) (ON685912) and the already published chloroplast genomes of *C*. *verum* (NC_035236.1, KY63578.1). However, the sample of *C*. *verum* from India (ON685911) differs in 387 positions from the sample from Sri Lanka, and in 384 positions from previously published *C*. *verum* chloroplast genomes in NCBI (NC_035236.1, KY63578.1). There are forty-one (41) polymorphic sites between chloroplast genomes of *C*. *ovalifolium* (ON685908) and *C*. *litseifolium* (ON685907). Interestingly, the variable sites between *C*. *verum* (India) (ON685911) and *C*. *ovalifolium* (ON685908) are 21. Furthermore, the variable sites between *C*. *verum* (India) (ON685911) and *C*. *litseifolium* (ON685907), *C*. *citriodorum* (ON685905), *C*. *capparu-coronde* (ON685904), and *C*. *sinharajaense* (ON685910) are 60, 159, 177 and 254 respectively. The variable sites between *C*. *rivulorum* (ON685909) and *C*. *dubium* (ON685906) are 86.

**Fig 2:**
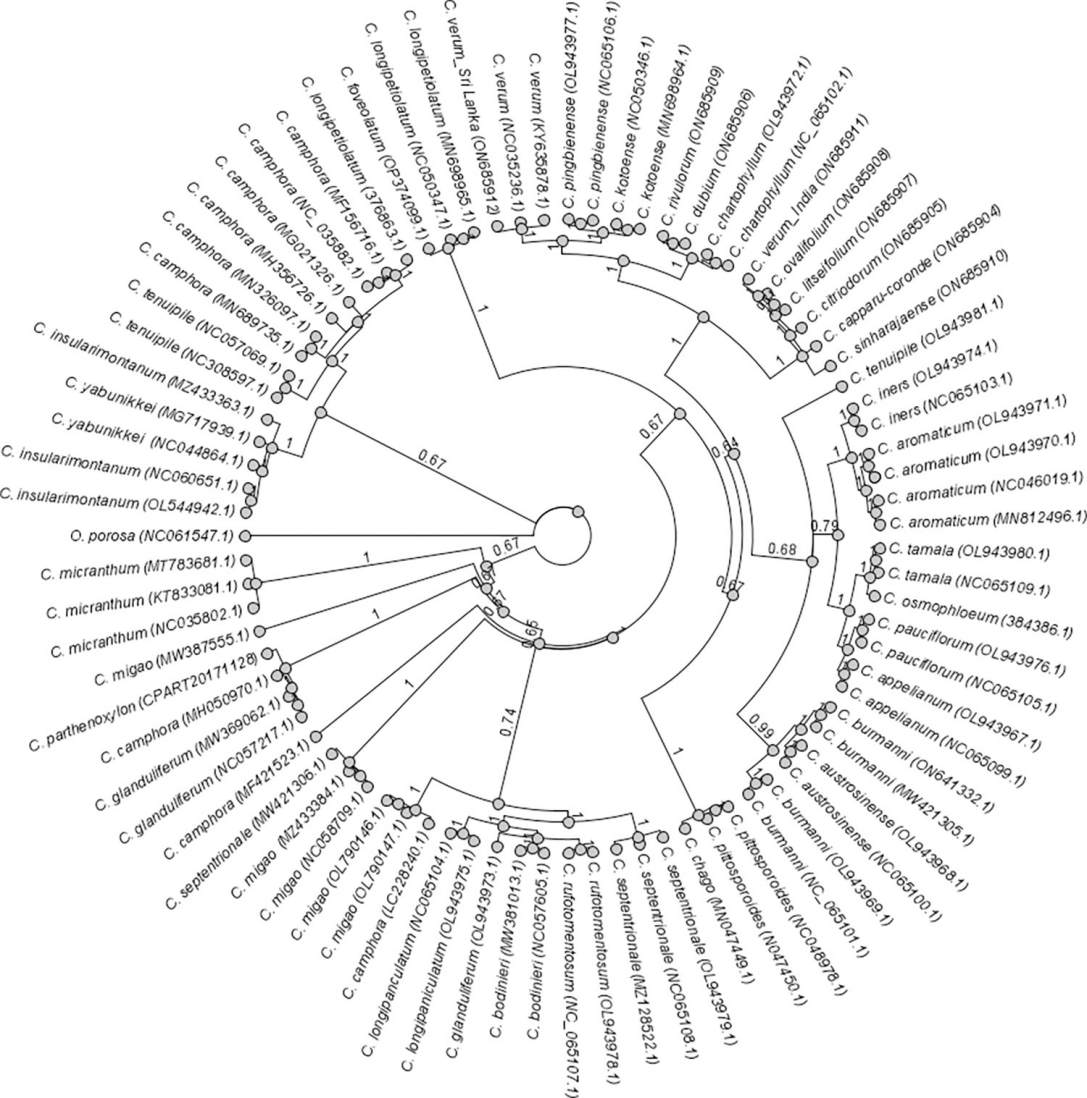
The Bayesian phylogenomic tree constructed for *Cinnamomum* species based on complete chloroplast sequences. Posterior probability values are given next to the nodes and *Ocotea porosa* was set as the outgroup.

The Bayesian phylogeny tree constructed with the complete chloroplast genomes of 83 samples is given (Fig 2). Most of the branches received the highest posterior probability (pp) value of 1, while the others ranged from 0.64 to 0.99. *Cinnamomum verum* (Sri Lanka) (ON685912) formed a monophyletic group with previously sequenced *C*. *verum* (NC_035236.1, KY635878.1), *C*. *pingbienense* (OL943977.1, NC065106.1), *C*. *kotoense* (NC050346.1, MN698964.1), *C*. *chartophyllum* (OL943972.1, NC_065102.1), *C*. *verum* (India) (ON685911) and all the wild *Cinnamomum* species in Sri Lanka (pp value 1). Within that monophyletic group there were two major sub-clades. The first sub clade includes *C*. *verum* (Sri Lanka) (ON685912), *C*. *verum* NCBI (NC_035236.1, KY635878.1), *C*. *pingbienense* (OL943977.1, NC065106.1), *C*. *kotoense* (NC050346.1, MN698964.1), *C*. *chartophyllum* (OL943972.1, NC_065102.1) and wild species of *C*. *rivulorum* (ON685909) and *C*. *dubium* (ON685906) (pp value 1) while *C*. *verum* (India) (ON685911), *C*. *ovalifolium* (ON685908), *C*. *litseifolium* (ON685907), *C*. *citriodorum* (ON685905), *C*. *capparu-coronde* (ON685904) and *C*. *sinharajaense* (ON685910) belongs to the second sub clade (pp value 1). In the first sub clade *C*. *verum* (Sri Lanka) (ON685912) was sister to *C*. *verum* NCBI (NC_035236.1, KY635878.1) with a pp value of 1, while *C*. *pingbienense* (OL943977.1, NC065106.1) was sistering to *C*. *kotoense* (NC050346.1, MN698964.1) with a pp value of 1. On the other hand, *C*. *rivulorum* (ON685909) and *C*. *dubium* (ON685906) were sister to *C*. *chartophyllum* (OL943972.1, NC_065102.1) with a pp value of 1. Within the second sub-clade *C*. *verum* (India) ON685911) was sister to *C*. *ovalifolium* (ON685908) and *C*. *litseifolium* (ON685907) with a pp value of 1.

### Nucleotide diversity analysis

**Fig 3:**
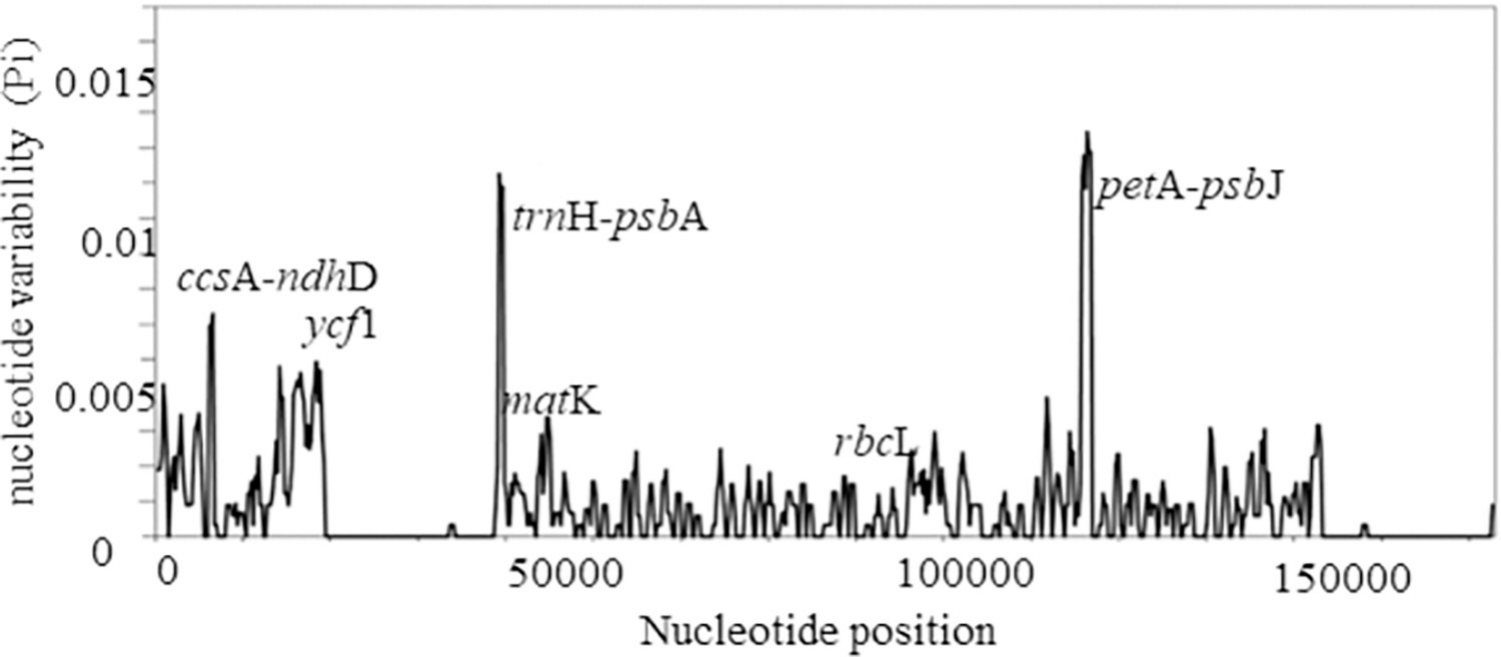
The nucleotide diversity (Pi) calculated for the chloroplast genomes of *Cinnamomum* species.

Nucleotide diversity analysis revealed two highly variable regions in the chloroplast genomes of the *Cinnamomum* species. Both were intergenic spacer regions, *trn*H-*psb*A, and *pet*A-*psb*J, with more than 0.01 nucleotide variability (Pi). The gene *ycf1* had the highest Pi value among genes. Universal barcoding genes, *mat*K and *rbc*L, had lower variability (Fig 3).

### Simple Sequence Repeat (SSR) analysis

Table 3 presents the distribution of SSR regions. Almost all the species have a similar SSR distribution. Mononucleotide microsatellites were the most abundant form of SSR, and A/T motifs were the most common among them. Trinucleotide microsatellites were the second most common SSR type, and the third were Dinucleotide repeats. The number of SSRs located outside the gene coding regions was double the number of SSRs located within the coding regions.

**Table 3:** SSR type, location in the genome, and the count among *Cinnamomum* species.

Species	SSR type						Location		
	Mono	Di	Tri	Tetra	Penta	Hexa	Non-coding	Coding	Total
*C*. *capparu-coronde* (ON685904)	130	36	68	8	1	1	160	84	244
*C*. *citriodorum* (ON685905)	130	36	68	8	1	1	160	84	244
*C*. *dubium* (ON685906)	132	38	69	8	1	1	164	85	249
*C*. *litseifolium* (ON685907)	131	36	68	8	1	1	160	85	245
*C*. *ovalifolium* (ON685908)	131	36	68	8	1	1	160	85	245
*C*. *rivulorum* (ON685909)	132	38	69	8	1	1	164	85	249
*C*. *sinharajaense* (ON685910)	130	36	69	8	1	1	160	85	245
*C*. *verum* (India) (ON685911)	131	36	68	8	1	1	156	89	245
*C*. *verum* (NC_035236)	130	38	68	8	1	1	161	85	246
*C*. *verum* (Sri Lanka) (NC685912)	130	38	68	8	1	1	157	89	246

### Diversity of Internal Transcribed Spacer (ITS) regions

Each assembled ITS sequence contains an 18S ribosomal RNA gene (rRNA) (Partial sequence), ITS 1, 5.8S rRNA, ITS 2, and 26S rRNA (partial sequence). ITS regions of *C*. *capparu-coronde*, *C*. *citriodorum*, *C*. *litseifolium*, *C*. *ovalifolium*, *C*. *sinharajaense*, and *C*. *verum* (India) are 599 bp in length, while it is 595 bp in *C*. *dubium*, *C*. *rivulorum*, and *C*. *verum* (Sri Lanka). The GC contents vary between 70%-71%.

The ITS region is the most variable region (Table 4) compared to the adjacent rDNA regions. Compared to the other species examined, *C*. *dubium*, *C*. *rivulorum*, *C*. *verum* NCBI, (SRX2990994), and *C*. *verum* (*S*ri Lanka) (OQ867307) have a 4 bp deletion in alignment positions 603–606. In the ITS 2 region, *C*. *dubium*, *C*. *rivulorum*, *C*. *verum* NCBI (SRX2990994), and *C*. *verum* (Sri Lanka) (OQ867307) share identical sequences, while another identical pattern is found in *C*. *capparu-coronde*, *C*. *citriodorum*, *C*. *litseifolium*, *C*. *ovalifolium*, *C*. *verum* (India) (OQ867448) and *C*. *sinharajaense*. Interestingly, *C*. *verum* Sri Lanka (OQ867307) and *C*. *verum* NCBI (SRX2990994) have only one (01) bp difference in the ITS region. *Cinnamomum verum* (India) (OQ867448) shares an identical ITS region with *C*. *ovalifolium*, *C*. *litseifolium*, *C*. *citriodorum*, and *C*. *capparu-coronde*. *Cinnamomum sinharajaense* has a single base difference at the 241^st^ alignment position compared to the group including *C*. *verum* (India) (OQ867448).

**Table 4:** Nucleotide diversity in ITS regions among 10 *Cinnamomum* species.

Species	Position																
	18S	ITS1							ITS2								
	1	78	112	160	161	171	218	241	494	546	561	566	593	603	604	605	606
*C*. *dubium* (OQ874796)	C	T	C	C	A	G	A	C	A	G	T	A	G	-	-	-	-
*C*. *rivulorum* (OQ888700)	C	T	C	C	A	G	A	C	A	G	T	A	G	-	-	-	-
*C*. *verum* NCBI (SRX2990994)	C	T	C	C	G	G	C	G	A	G	T	A	G	-	-	-	-
*C*. *verum* (Sri Lanka) (OQ867307)	C	T	C	C	G	G	C	T	A	G	T	A	G	-	-	-	-
*C*. *capparu-coronde* (OQ888687)	T	C	T	A	A	A	C	T	T	A	C	G	A	C	C	A	T
*C*. *citriodorum* (OQ874734)	T	C	T	A	A	A	C	T	T	A	C	G	A	C	C	A	T
*C*. *litseifolium* (OQ874733)	T	C	T	A	A	A	C	T	T	A	C	G	A	C	C	A	T
*C*. *ovalifolium* (OQ888686)	T	C	T	A	A	A	C	T	T	A	C	G	A	C	C	A	T
*C*. *verum* (India) (OQ867448)	T	C	T	A	A	A	C	T	T	A	C	G	A	C	C	A	T
*C*. *sinharajaense* (OQ867450)	T	C	T	A	A	A	C	C	T	A	C	G	A	C	C	A	T

The distance matrix obtained for the multiple sequence alignments of ITS regions of nine *Cinnamomum* samples sequenced in this study and the NCBI samples is given in S4 Table. The number of variable sites ranged from 0 to 325. There was no variation between *C*. *verum* (Sri Lanka) (OQ867307) and three sequences of *C*. *verum* from NCBI (KU139902, KU139903, KX766399), while there was a single base pair difference between *C*. *verum* (Sri Lanka) (OQ867307) and another five sequences from NCBI (SRX2990994, MF110059, MF110060, MF110061, and KX509827). In contrast, there are twenty (20) variable sites between *C*. *verum* (Sri Lanka) (OQ867307) and *C*. *verum* (India) (OQ867448). Variable sites between *C*. *verum* (Sri Lanka) (OQ867307) and wild *Cinnamomum* species from Sri Lanka ranged from 3 to 21, where the difference between *C*. *verum* (Sri Lanka) (OQ867307) compared to *C*. *dubium* (OQ874796) and *C*. *rivulorum* (OQ888700) was three (3), and it was twenty (20) compared to *C*. *capparu-coronde* (OQ888687), *C*. *citriodorum* (OQ874734), *C*. *litseifolium* (OQ874733) and *C*. *ovalifolium* (OQ888686), and twenty-one (21) compared to *C*. *sinharajaense* (OQ867450*)*. Interestingly, the number of variable sites between *C*. *verum* (India) (OQ867448) and wild *Cinnamomum* species from Sri Lanka ranged from 0 to 21 as well. There was no difference between *C*. *verum* (India) (OQ867448) and *C*. *capparu-coronde* (OQ888687), *C*. *citriodorum* (OQ874734), *C*. *litseifolium* (OQ874733), and *C*. *ovalifolium* (OQ888686), while there was one nucleotide difference compared to *C*. *sinharajaense* (OQ867450*)*. *Cinnamomum verum* India (OQ867448) differs from the wild species *C*. *dubium* (OQ874796) and *C*. *rivulorum* (OQ888700) in twenty-one (21) base pairs.

**Fig 4:**
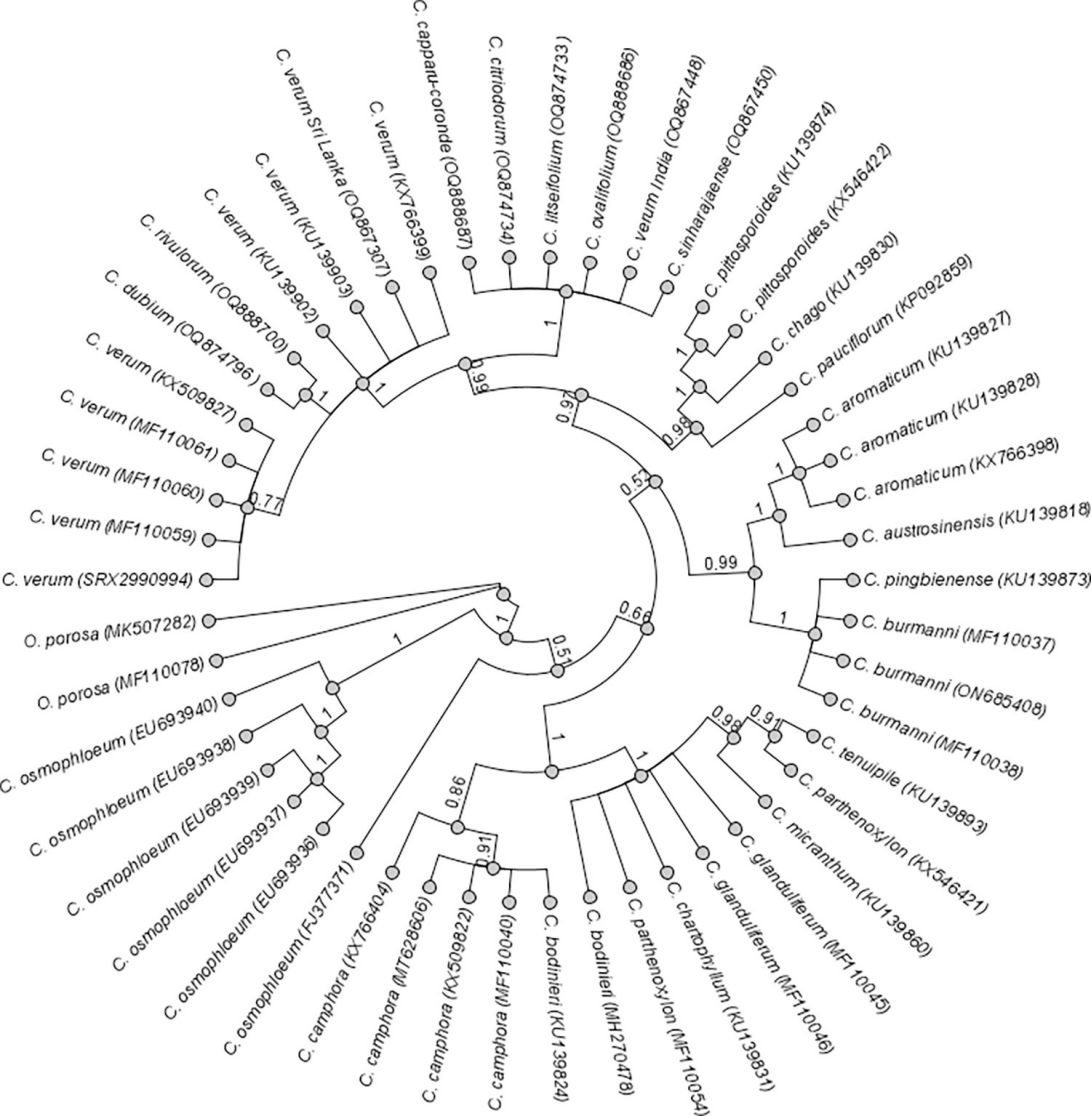
The Bayesian phylogenomic tree constructed for *Cinnamomum* species based on ITS regions. Posterior probability values are given next to the nodes and *Ocotea porosa* was set as the outgroup.

The Bayesian phylogenomic tree constructed for ITS sequences of 50 *Cinnamomum* samples show taxa relationship (Fig 4). *Cinnamomum verum* (Sri Lanka) (OQ867307) formed a monophyletic group with *C*. *capparu-coronde* (OQ888687), *C*. *citriodorum* (OQ874734), *C*. *litseifolium* (OQ874733), *C*. *ovalifolium* (OQ888686), *C*. *verum* India (OQ867448), *C*. *sinharajaense* (OQ867450), *C*. *dubium* (OQ874796), *C*. *rivulorum* (OQ888700), *C*. *verum* NCBI (SRX2990994, MF110059, MF110060, MF110061, KX509827, KU139902, KU139903, KX766399) with pp value of 0.99. There were two major sub clades within that monophyletic group. *Cinnamomum verum* (Sri Lanka) belongs to the first sub-clade with eight *C*. *verum* NCBI samples (SRX29990994, MF110059, MF110060, MF110061, KX509827, KU139902, KU139903, KX766399) and two wild species *C*. *dubium* (OQ874796) and *C*. *rivulorum* (OQ888700) (pp value of 1). Among the *C*. *verum* samples, SRX299094, MF110059, MF110060, MF110061 and KX509827 were closer (pp value 0.77), compared to *C*. *verum* samples of KU139902, KU139903, KX766399 and *C*. *verum* Sri Lanka (OQ867307). *Cinnamomum verum* (India) (OQ867448) and the remaining wild species of *C*. *capparu-coronde* (OQ888687), *C*. *citriodorum* (OQ874734), *C*. *litseifolium* (OQ874733), *C*. *ovalifolium* (OQ888686) and *C*. *sinharajaense* (OQ867450) belong to the second subclade (pp value 1*)*.

### Mitochondrial genes–assembly and analysis

Assembly of the complete plant mitochondrial genome is challenging [72]. Therefore, we looked at several regions *atp6* (631 bp), *cox1*(1415 bp), *atp1* (1262 bp), *matR* (1777 bp), and *atpA* (1239 bp) of the mitochondrial genome. Among them, *atp1* (1530 bp), *matR* (1777 bp), and *atpA* (1239 bp) regions were identical in the species examined, while *atp6* (631bp) had nine, and cox*1* (1415 bp) had one variable site. While SNPs in the *atp6* gene cause 4 synonymous and 5 nonsynonymous changes, the SNP in the *cox1* gene is synonymous (S5 Table). Interestingly, *C*. *verum* (Sri Lanka), *C*. *verum* NCBI (SRX2990994), and *C*. *dubium* share an indel region between 766 and 771 bp and share identical *atp6* genes and proteins.

**Fig 5:**
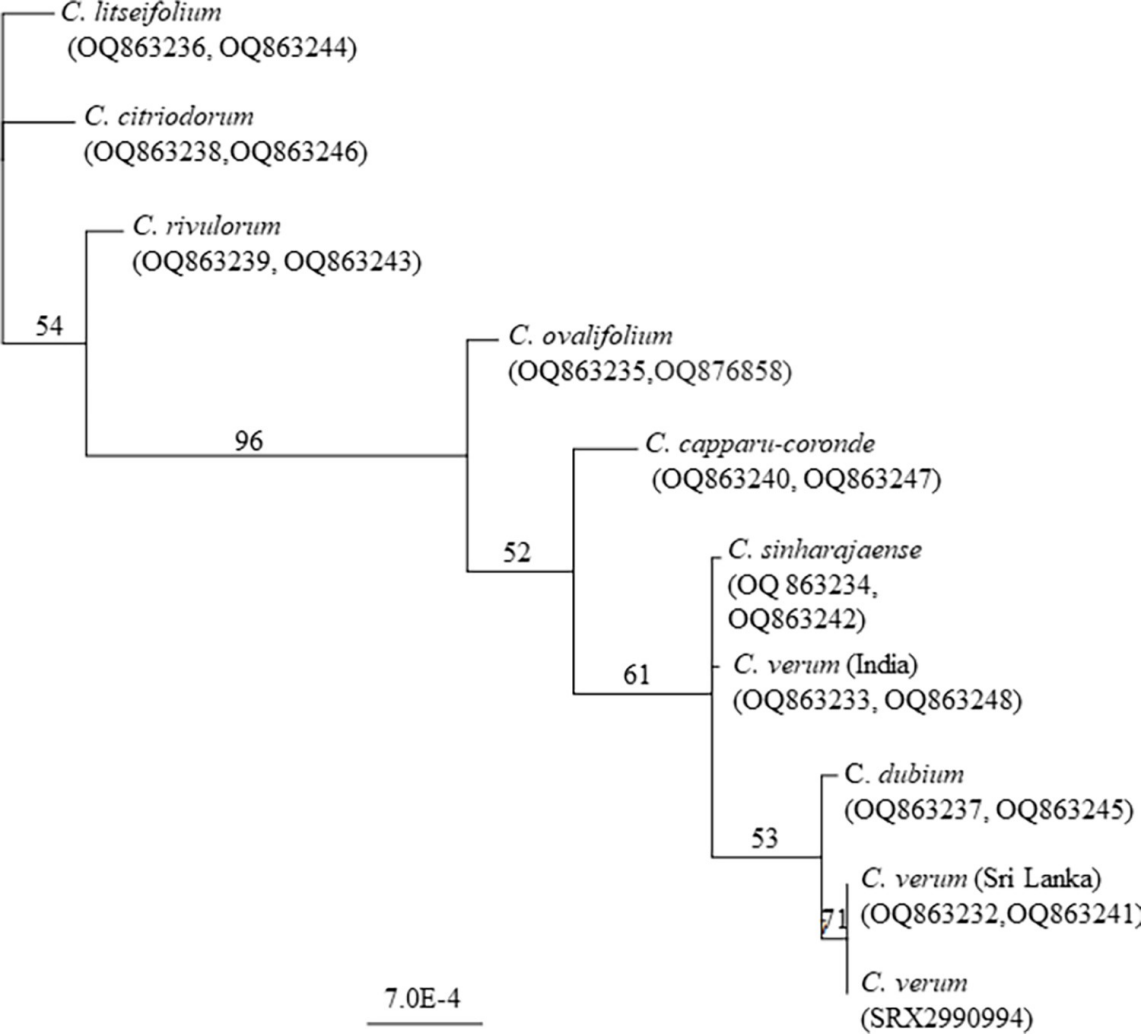
The unrooted Neighbor Joining Consensus tree constructed for *Cinnamomum* species based on mitochondrial *atp6* and cox*1* genes. Bootstrap values are given next to the nodes. GenBank accession numbers of *atp6* (631 bp) and cox*1* (1415 bp) genes are given respectively next to the species names.

According to the Neighbor-Joining Consensus tree constructed for combined datasets of *atp6* (631 bp) and cox*1* (1415 bp) genes *C*. *verum* (India) (OQ863233,OQ863248), *C*. *ovalifolium* (OQ863235,OQ876858) *C*. *dubium* (OQ863237,OQ863245) *C*. *capparu-coronde* (OQ863240 OQ863247), *C*. *sinharajaense* (OQ863234,OQ863242), *C*. *verum* (Sri Lanka) (OQ863232, OQ863241) *C*. *verum* NCBI (SRX2990994) and *C*. *verum* (India) (OQ863233, OQ863248) clustered together (bootstrap 96) while *C*. *verum* (Sri Lanka) (OQ863232, OQ863241) and *C*. *verum* NCBI (SRX2990994) further clustering together (bootstrap value 71) (Fig 5).

### Skmer analysis

**Fig 6:**
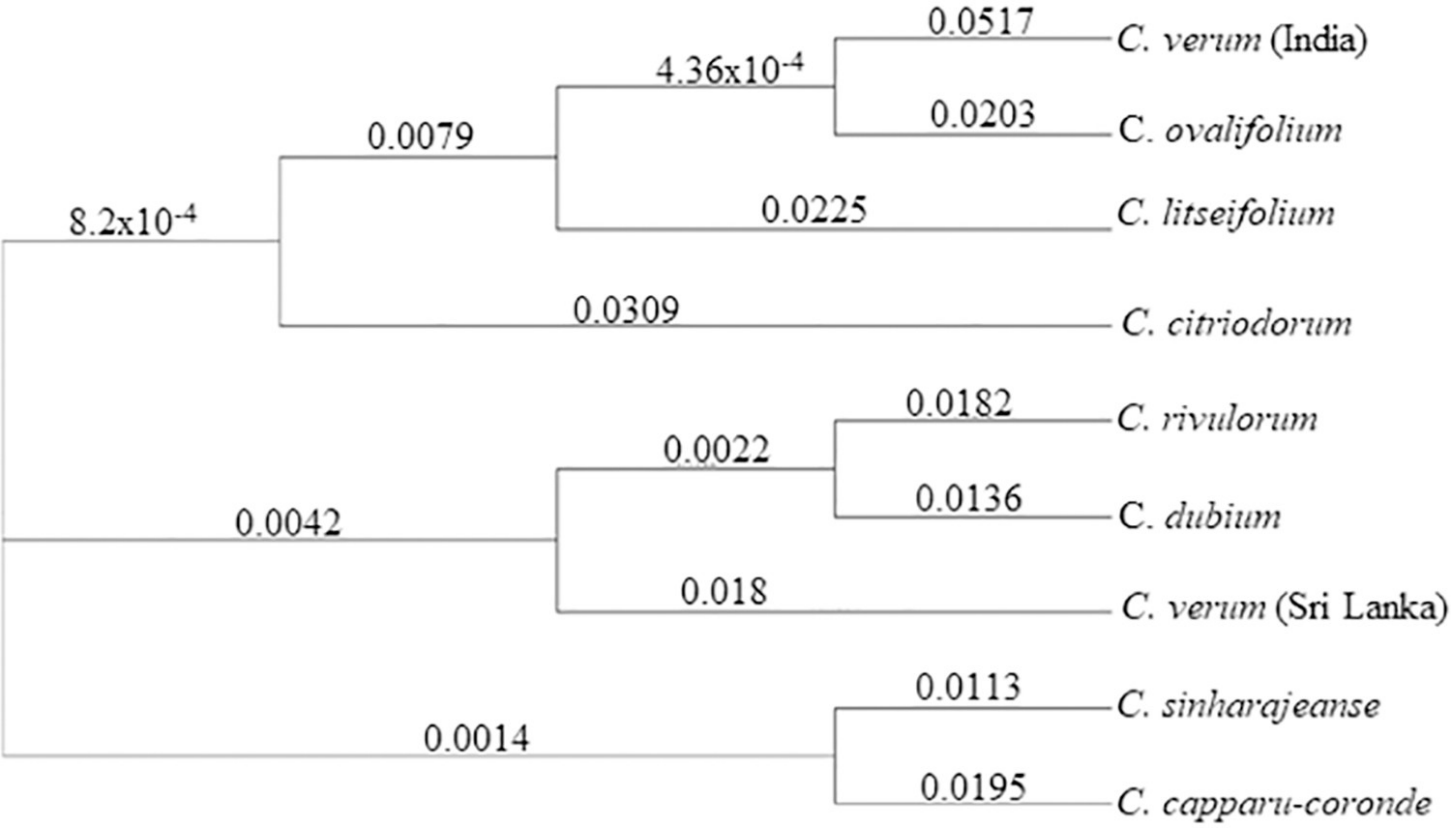
The phylogenetic tree drawn using the distance matrix obtained from Skmer v3.0.2. Bootstrap values are given next to the nodes.

Interestingly, Skmer V 3.02 grouped Sri Lankan *Cinnamomum* species into three clades (Fig 6). *Cinnamomum verum* (Sri Lanka) is sister to C. *dubium* and *C*. *rivulorum* with a bootstrap value of 0.0042, whereas *C*. *verum* (India) is sister to *C*. *ovalifolium*, *C*. *litseifolium* and *C*. *citriodorum* (8.2x10^-4^). Furthermore, *C*. *sinharajaense* and *C*. *capparu-coronde* formed a clade with bootstrap value of 0.0014.

### Primer design, PCR, Sanger sequencing and data analysis

Since *C*. *verum* (India) and *C*. *verum* (Sri Lanka) had considerable differences in all the analyses, we analyzed more samples from these two groups. Additional eight *C*. *verum* (Sri Lanka) accessions showing considerably different morphological and chemical traits [16], and two *C*. *verum* (India) were assessed with the most variable regions (*ycf1* gene) in the chloroplast genome. The two regions of the *ycf1* gene were PCR amplified, and all the samples resulted in good-quality sequencing data. The alignment included the same regions as extracted from Illumina data and already deposited in GenBank (S6 Table). The first region of the *ycf1* gene has thirteen variable sites among the three *C*. *verum* samples from India and the examined samples from Sri Lanka including wild species. The second *ycf1* region includes seventeen variable sites and an indel region of 18 bp. Altogether, 30 variable sites and an indel region were found among the examined species. While the variable sites vary among samples, all the *C*. *verum* (Sri Lanka) samples studied show an 18 bp insertion in the second *ycf1* region, which is not present in three samples collected from India. The same insertion is present in *C*. *verum* NCBI (NC_035236) and *C*. *sinharajaense*, while it is absent in all the other Sri Lankan wild species and *C*. *aromaticum* (NC046019).

Furthermore, sequence divergence calculated for combined sequences of each group revealed that there is no within-group sequence divergence for *C*. *verum* (Sri Lanka), while it is 0.006 for *C*. *verum* (India). The intergroup sequence divergence for *C*. *verum* (Sri Lanka) and *C*. *verum* (India) is 0.007. As such, when considering *C*. *verum* (Sri Lanka) and *C*. *verum* (India) as two groups, the sequence divergence between them is higher than the within-group sequence divergence of each species.

## Discussion

In this study we assembled the chloroplast genomes, ITS regions, *atp6*, *cox1*, *atp1*, *matR*, and *atpA* of the mitochondrial genome and Skmer analysis using the 20x coverage Illumina Hiseq data from *Cinnamomum* species found in Sri Lanka. We did similar analyses using all the datasets and gene regions for easy comparisons. Interestingly, all the analyses supported a similar pattern of evolutionary relationship among *Cinnamomum* species in Sri Lanka. The picture is clearer than what we observed with the universal barcoding regions [25].

There are about 100 chloroplasts in typical mesophyll cells of plants such as *Arabidopsis*, wheat, and rice [73–75]. Regular DNA extraction protocols such as CTAB [76] and SDS [77] result in total cellular DNA, including genomic, chloroplast, and mitochondrial DNA. However, there are protocols available for additional enrichment of chloroplast DNA, including plastid isolation, enrichment via methylation-sensitive capture, hybrid bait capture, and PCR [78,79]. Nevertheless, these isolation or enrichment procedures are time-consuming and expensive [80]. Therefore, “skim sequencing” has become common now, in which the total DNA is sequenced and the chloroplast DNA is separated bioinformatically [80,81]. This approach is more cost-effective, as a total genome sequenced at a lower coverage usually results in sufficient coverage of chloroplast DNA for assembling the chloroplast genome. It is suggested that a sequencing coverage of ~0.1 – 10x for the nuclear genome is sufficient for the genome skimming approach [82,83].

When assembling complete chloroplast genomes from total DNA, it is vital to identify the most effective assembly method and bioinformatics tools to obtain the highest accuracy in results. Even degraded herbarium material has been successfully assembled with genome skimming, but it is necessary to give special attention to the assembly process [84]. When sequencing, a suitable platform that provides read lengths larger than repeat lengths in the plastome should be chosen. Currently, most NGS platforms fulfill this requirement, and the reads generated are sufficient for *de novo* assemblies [85]. A coverage of 30x and more than 500 Mb of sequencing data is considered sufficient to generate a good quality assembly [82]. Hence, Illumina HiSeq provides a cost-effective solution and high throughput for larger genome skimming.

When performing genome skimming to assemble a chloroplast genome, it is generally a good idea to first separate the chloroplast reads from nuclear DNA before assembly. It reduces the complexity of the data, aiding the *de novo* assembly process [82]. Among suitable *de novo* assemblers for filtered chloroplast reads are Geneious [48], MIRA [86], ABySS [87], SOAPdenovo [88], SPAdes [58], and Velvet [89]. Some assemblers such as MITObim [90], Fast-plast [91], and NOVOPlasty [45] merge filtering of chloroplast reads and assembly processes. These assemblers use a known plastid sequence as a seed or a ‘bait’ to identify chloroplast reads within total DNABased on the assembly statistics. We considered the seed-based assemblies to be more reliable to continue with the annotation and analysis. The amount of data were sufficient to assemble the ITS regions, 18S ribosomal RNA gene (rRNA) (Partial sequence), ITS 1, 5.8S rRNA, ITS 2, and 26S rRNA (partial sequence). While the data generated were not sufficient to assemble the mitogenomes, several mitochondrial regions were assembled and included in the analysis.

We encountered several bioinformatics-related challenges during the optimization process. For example, whether sufficient coverage of data could be generated to assemble chloroplast sequences from dry bark. Based on the chloroplast analysis, it was clear that the data is comparable to the data generated from green leaves. For example, *C*. *verum* (India) has the smallest N50 value among the nine (9) chloroplast assemblies. This behavior in *C*. *verum* (India, ON685911) data could also be due to the sample being a bark. As barks do not contain much chloroplast DNA, the amount of nuclear DNA could be high within the sequenced data of *C*. *verum* (India, ON685911). This could potentially be a challenge for the *de novo* assembler and would provide a large number of contigs with more gaps than leaf samples. Similarly, a higher nuclear DNA amount could have affected the N50 value of *C*. *verum* (India, ON685911) being lower than that of other assemblies. Further, if a high repeat content is present, it could also affect the N50 value as the assembler would struggle to produce longer contigs.

However, since the *C*. *verum* (India, ON685911) assembly length, amount of chloroplast reads assembled, and GC content is in range with all other assemblies, it can be considered of good quality. In addition, the percentage of chloroplast reads assembled in *C*. *ovalifolium* is even smaller than the assembled chloroplast reads of *C*. *verum* (India, ON685911). Nevertheless, the *C*. *ovalifolium* assembly is of very good quality considering the N50 value. This indicates that the available chloroplast reads in the total DNA were sufficient to assemble a good quality *C*. *verum* (India, ON685911) chloroplast genome. The assembled chloroplast genomes were submitted to NCBI GenBank.

According to the complete chloroplast analysis, *C*. *chartophyllum* (158 kb) had a larger genome size compared with published chloroplast genomes and the other nine newly sequenced chloroplast genomes in this study. The authors, Ge *et al*. 2022, predicted that the larger size of *C*. *chartophyllum* is due to the IR expansion, resulting in duplication of complete *trnICAU*, *rpl32*, *rpl2*, *and ycf2* in the IR regions, which was the first case in the genus *Cinnamomum* [88]. Nevertheless, it might also be due to artefacts of the *de novo* plastome assembly process. Surprisingly, *C*. *verum* (Sri Lanka) and *C*. *verum* (NC_035236.1, KY635878.1) are more closely related to *C*. *pingbienense* (OL943977.1, NC65106.1) and *C*. *kotoense* (NC050346.1, MN698 964.1) than to wild *Cinnamomum* samples in Sri Lanka and *C*. *verum* (India). *Cinnamomum kotoense* is an endangered species in Lanyu island, Taiwan, and is an ornamental plant. It is reported that *C*. *kotoense* is closely related to *C*. *verum* and *C*. *aromaticum* [92]. *Cinnamomum pingbienense* is native to South-Central and Southeast China [93]. The branch support values are high (pp value1), confirming the accuracy of clustering based on chloroplast data deposited on the NCBI. However, further analysis and studying type specimens will be needed to confirm such relationships.

In our previous analyses, nucleotide diversity in *rbc*L, *mat*K and *trn*H-*psb*A regions was not sufficient to differentiate *C*. *verum* (Sri Lanka) from *C*. *sinharajaense*, or *C*. *litseifolium* from *C*. *ovalifolium* and *C*. *citriodorum* [25]. The current analysis suggests comparatively higher nucleotide diversity in the *trn*H-*psb*A and *pet*A-*psb*J regions than in the common universal barcoding regions. However, a previous study has also reported inadequacy of the nucleotide diversity in the *trn*H-*psb*A region for molecular level identification of the *Cinnamomum* species in Sri Lanka [5]. The current work proposes new barcode regions of *ycf1*, ITS and mitochondrial genes of *atp6* and cox*1* for the identification of *Cinnamomum* species.

Complete chloroplast and ITS analyses suggest that *C*. *verum* (Sri Lanka) is more closely related to *C*. *verum* samples in NCBI, *C*. *dubium* and *C*. *rivulorum* than to other species. Further, *C*. *ovalifolium* and *C*. *litseifolium* always group together suggesting DNA level similarity between them. Interestingly, *C*. *verum* (India, ON685911) groups with *C*. *ovalifolium* and *C*. *litseifolium* but not with *C*. *verum* (Sri Lanka) (or at least not immediately). Further *C*. *litseifolium* is only found in restricted habitats above 1800 m elevation [8]. However, the sequences retrieved from NCBI of specimens identified as *C*. *verum* share an identical *atp6* region (SRX299099), an identical *Cox1* region (SRX299099) and a single base pair mismatch in the ITS region with *C*. *verum* (Sri Lanka, OQ867307). While most of the chloroplast regions are identical there is a 5 bp mismatch in the complete chloroplast genomes between *C*. *verum* (Sri Lanka, OQ685912) and *C*. *verum* (NC_035236). We observed the same with our previous work on barcoding regions [25]. Therefore, the available chloroplast genome (ON685912) and the rest of the sequences of *C*. *verum* in the NCBI (NC_035236.1, KY635878.1) could have originated from Sri Lanka because some countries specially China and India grow Ceylon cinnamon.

The NCBI consisted of ninety complete chloroplast genome assemblies and more than 250 ITS regions that we could include in the analysis. However, there was no sufficient data for mitochondrial gene regions, except *C*. *chekiangense*. Similarly, we limited Skemer analysis to our dataset since there was limited skim sequencing data for the genus *Cinnamomum*. Therefore, the number of taxa in each analysis and the topology of phylogenetic tree were not comparable. However, relationship among samples included in this study were consistent.

The wild populations of *C*. *verum* (Sri Lanka) are still found in the upcountry and mid-country rain forests in Sri Lanka [13,20,94]. Such findings support the historical evidence of the origin of cultivated Cinnamon, where Portuguese and Dutch invaders started commercial cultivation in the southern part of the country. The historical evidence suggests the introduction of *C*. *verum* to India by taking a bunch of seeds from Sri Lanka in the 1920s [10]. Therefore, a closer relationship is expected between *C*. *verum* (Sri Lanka) and *C*. *verum* (India). However, all the above analyses suggested they are further apart when considering DNA diversity. The morphology [13], molecular [25], and biochemical [17] data suggest intraspecies diversity in both cultivated and wild *Cinnamomum* species in Sri Lanka. We selected a group of *C*. *verum* samples identified as most diverse in morphological, biochemical, and yield-related traits from our previous work to assess their molecular diversity and to compare them with the samples collected from India. PCR primers were designed for the *ycf1* gene, the most variable region of the chloroplast genome, that we identified in the current study. *The ycf*1 gene, a hypervariable region, is the most variable locus and achieved better phylogenomic resolutions than standard DNA barcodes in land plants for phylogenomic studies [95,96]. They also suggest it as a cost-effective method, considering that complete chloroplast genome sequencing requires high-quality DNA, a higher cost for sequencing, and bioinformatics facilities.

There is considerable intraspecies diversity among *C*. *verum* (India, NC_035236.1,) as well as in *C*. *verum* (Sri Lanka, ON685912) for *ycf1*. However, the indel region between position 736 and 753 is conserved in all nine samples of *C*. *verum* (Sri Lanka), where the 18 bp motif (GTCCCTATAGAATCTTCT) is duplicated. The same sequence is present in a *C*. *verum* chloroplast genome (NC_035236.1) deposited in NCBI and in *C*. *sinharajaense* (ON685910). Only one copy of the 18 bp region is present in all the other *Cinnamomum* species in Sri Lanka as well as in the three *C*. *verum* samples from India. Therefore, sequence data in NCBI might be linked to Sri Lanka, though the authors have purchased it from an ornamental plant grower, Top Tropicals and the authenticity information has not been mentioned [60]. *Cinnamomum sinharajaense* is only found in restricted locations of the Sinharaja rainforest and is considered threatened. Therefore, there is a minimal possibility of appearing *C*. *sinharajaense* in either local or foreign markets. Therefore, this indel region is useful as a marker to differentiate *C*. *verum* (India) from *C*. *verum* (Sri Lanka). Further, there was no nucleotide diversity in the *ycf1* region among *the C*. *verum* (Sri Lanka) collections included in the analysis while it was 0.006 among *the C*. *verum* (India) collection. However, the diversity between *C*. *verum* samples collected from India and Sri Lanka is higher than the intra-sample diversity values.

## Conclusions

All the analysis suggests that *C*. *verum* chloroplast sequencing data (NC_035236.1) deposited in the NCBI database could be from a sample of Sri Lankan origin. Chandrasekara *et al*. 2021 suggested the same based on an analysis of universal barcoding regions [25]. Interestingly, *C*. *verum* (Sri Lanka) and *C*. *sinharajaense* have similar chemical profiles except for differences in the relative abundance of some compounds [94]. While *C*. *sinharajaense* can be identified morphologically, there is no evidence for morphological differences between *C*. *verum* from Sri Lanka and *C*. *verum* from India. While the name *C*. *zeylanicum* [97] has widely been used in the literature for Sri Lankan Cinnamon, it is considered a synonym for *C*. *verum* [98]. However, the Scanning Electron Microscopic analysis (SEM) of *C*. *verum* (Sri Lanka) pollen samples [5] suggests considerable differences compared to recently published SEM data of *C*. *verum* (India) [99]. The pollen size and the spine length are different between them. Such differences are to be studied comprehensively. Nevertheless, the molecular data presented here would provide complementary evidence. Although this analysis included only a single accession from each species, it consisted of the complete chloroplast genome, a considerable region in the mitochondrial genome, and many coding and non-coding regions of the nuclear genome. Individual regions and combined analyses were conducted and resulted in similar evolutionary relationships and associations. Therefore, the robustness of the data and the analysis are confirmed, similar to our previous work on transcriptomics [100]. We propose this as a cost-effective analysis method for studying phylogenomic relationships among closely related species.

## Supporting information

S1 Table Details of the wild *Cinnamomum* samples collected. (DOCX)

S2 Table Features of the chloroplast genome. (XLSX)

S3 Table Distance matrix obtained for the chloroplast genome alignment of *Cinnamomum* samples. (XLSX)

S4 Table Distance matrix obtained for the ITS alignment of *Cinnamomum* samples. (XLSX)

S5 Table Nucleotide diversity of the mitochondrial genes *atp6* and *cox1* among 10 *Cinnamomum* species. Accession numbers in the first column represent *atp6* and *cox1* respectively.
(XLSX)

S6 Table Sequence variation in two parts of the *ycf1* gene among *Cinnamomum* species. (XLSX)
