# Supplementary material for: Chloroplast genome, nuclear ITS regions, mitogenome regions, and Skmer analysis resolved the genetic relationship among Cinnamomum species in Sri Lanka
Source: PLoS One. 2023 Sep 20;18(9):e0291763. doi: 10.1371/journal.pone.0291763 (PMC10511092; doi:10.1371/journal.pone.0291763)
Supplement: S1 Table — (DOCX) [file pone.0291763.s001.docx]

| **Species** | **District** | **Location** | **Collected and identified by** | **Confirmed by** | **Voucher. No** |
| --- | --- | --- | --- | --- | --- |
| *C. capparu-coronde* | Matara | N-6.0263 E-80.5616 | K.G.G Wijesinghe,  Supun Bandusekara | Prof D. Siril A. Wijesundera^*^ | KGG.BS-2018-8-CC-M-1 |
| *C. citriodorum* | Matara | N-6.0225 E-80.5623 | K.G.G Wijesinghe,  Supun Bandusekara | Prof D. Siril A. Wijesundera | KGG.BS-2018-8-C-M-1 |
| *C. dubium* | Rathnapura | N-6.4108 E-80.5088 | Dr. R.H.G. Ranil,  Supun Bandusekara | Prof D. Siril A. Wijesundera | RHG.BS-2018-11-D-S-1 |
| *C. litseifolium* | N’eliya | N-6.9683 E-80.7700 | Prof. D. Siril A. Wijesundera, Prof. P.C.G. Bandaranayake,  Supun Bandusekara | Prof D. Siril A. Wijesundera | DSA.PCG.BS-2018-5-L-H-1 |
| *C. ovalifolium* | N’eliya | N-6.9696 E-80.7700 | Prof D. Siril A. Wijesundera, Prof. P.C.G. Bandaranayake,  Supun Bandusekara | Prof D. Siril A. Wijesundera | DSA.PCG.BS-2018-5-O-H-1 |
| *C. rivulorum* | Matara | N-6.0222 E-80.5624 | K.G.G Wijesinghe,  Supun Bandusekara | Prof D. Siril A. Wijesundera | KGG.BS-2018-8-R-M-1 |
| *C. sinharajaense* | Rathnapura | N-6.4352 E-80.4197 | Dr R.H.G. Ranil,  Supun Bandusekara | Prof D. Siril A. Wijesundera | RHG.BS-2018-11-S-S-1 |

^*^Taxonomist
